# The Variation Analysis of DNA Methylation in Wheat Carrying Gametocidal Chromosome 3C from *Aegilops triuncialis*

**DOI:** 10.3390/ijms18081738

**Published:** 2017-08-10

**Authors:** Dan Wang, Jieyu Zhao, Yan Bai, You Ao, Changhong Guo

**Affiliations:** Key Laboratory of Molecular Cytogenetics and Genetic Breeding of Heilongjiang Province, College of Life Science and Technology, Harbin Normal University, No. 1 of Shida Road, Limin Development Zone, Harbin 150025, China; wangdan19881234@163.com (D.W.); zhaojieyu8908@126.com (J.Z.); baiyan789@163.com (Y.B.); aoyou6@163.com (Y.A.)

**Keywords:** gametocidal chromosomes, common wheat, DNA methylation, MSAP (methylation sensitive amplified polymorphism), transposons

## Abstract

Gametocidal (Gc) chromosomes can ensure their preferential transmission by killing the gametes without themselves through causing chromosome breakage and therefore have been exploited as an effective tool for genetic breeding. However, to date very little is known about the molecular mechanism of Gc action. In this study, we used methylation-sensitive amplified polymorphism (MSAP) technique to assess the extent and pattern of cytosine methylation alterations at the whole genome level between two lines of wheat Gc addition line and their common wheat parent. The results indicated that the overall levels of cytosine methylation of two studied Gc addition lines (CS–3C and CS–3C3C, 48.68% and 48.65%, respectively) were significantly increased when compared to common wheat CS (41.31%) and no matter fully methylated or hemimethylated rates enhanced in Gc addition lines. A set of 30 isolated fragments that showed different DNA methylation or demethylation patterns between the three lines were sequenced and the results indicated that 8 fragments showed significant homology to known sequences, of which three were homologous to MITE transposon (Miniature inverted–repeat transposable elements), LTR-retrotransposon *WIS-1p* and retrotransposon *Gypsy*, respectively. Overall, our results showed that DNA methylation could play a role in the Gc action.

## 1. Introduction

Gametocidal (Gc) factors are strong segregation distorters that can affect plant fertility by killing gametes that do not contain them [1,2,3]. They were discovered in the process of the production of alien chromosome addition lines and alloplasmic lines from interspecific hybridization and backcrossion of common wheat *Triticum aestivum* L. (2*n* = 6x = 42, AABBDD) with wild *Aegilops* species [1,2]. When the wheat lines are hetero- or hemizygous for a Gc chromosome, the first and second meiotic divisions were cytologically normal with the chromosome configuration at metaphase I was: 2*n* = 21 II + 1 I, however, Gc chromosome can kill the gametes that do not contain it via the induction of chromosome breakage to ensure its preferential transmission during the first post-meiotic mitosis interphase in the hybrid progeny; however, no chromosome breakage appears in homozygotes for the Gc gene (that is, disomic alien chromosome addition lines) [2,4,5]. This genetic phenomenon is considerable interesting and some breeders have used Gc chromosome as an effective tool in genetic breeding [6]. Currently, Gc chromosomes have been widely utilized for the production of deletion stocks in common wheat and the rearrangement of alien chromosomes added to common wheat, which is greatly significant for making use of genetic resources of wheat and its related species to resist disease and breed high-quality wheat. So far, the Gc system has been used to create Gc chromosome translocation and deletion lines of wheat-barley, wheat-rye, wheat-*Elytrigia elongata* and wheat-*Agropyron* [6,7,8,9]. However, to date very little is known about the molecular mechanism of Gc action.

Gc chromosomes share with a common mechanism of action, which could induce frequent chromosomal structural rearrangements as well as multiple forms of chromosome aberration [10]. However, the mechanism of Gc chromosome is still unclear so far. Several hypotheses have been proposed to provide a mechanism for Gc action, including the famous dual-function model proposed by Tsujimoto, in which Gc genes have both the “breaking” and “protecting” functions [1,11]. When a Gc gene is transferred to the wheat genome, it produces two kinds of enzyme that one is a restriction enzyme (RE) which cleaves the specific restriction sites that it recognizes and the other is a modification enzyme (ME) like a methylase, which can protect restriction sites from being cleaved by RE [1,11]. This model can explain chromosome breakage in the gametes without Gc chromosome in the hemizygote of Gc gene and account for the reason why no chromosome breakage appears in homozygotes for the Gc gene [11]. Although this model can explain the dual-function of Gc chromosome, the investigator did not provide powerful experimental evidence to support it. In plants, various pattern of cytosine methylation is regulated and maintained by diverse methylases [12]. Some studies have reported that the DNA hypomethylating agent 5-azacytidine (5-AC) induced chromosome fragmentation in the root tips cells of wheat carrying the Gc chromosome, which suggested that DNA methylation can repress chromosome breakages induced by the Gc factors [13,14].

DNA cytosine methylation as one of the main epigenetic mechanisms in higher eukaryotes can play a vital role in maintaining genome stability and regulating gene expression [15,16]. In plants, DNA methylation can occur in all the sequence contexts of cytosine, including symmetric CG, CHG and asymmetric CHH (where H stands for A, C, or T) [17,18]. Both the symmetric CG and CHG methylation can be maintained during DNA replication, however, CHH methylation must be reestablished by RNA-directed DNA methylation (RdDM) [16,19]. DNA methylation is associated with numerous biological processes, including genomic imprinting, silence of transposable elements and regulation of gene expression [20,21,22]. It has been shown that DNA methylation may be involved in flower development and the sterility-fertility transition in plants [23,24]. Methylation-sensitive amplified polymorphism (MSAP), a technique for detecting the levels and patterns of genome DNA methylation, has been widely used in plant [25,26,27,28,29]. MSAP is based on the amplified fragment length polymorphism (AFLP) technology and takes advantage of two isoschizomers, *Msp* I and *Hpa* II, which can recognize the same restriction site (5′-CCGG-3′) but display differential sensitivity to DNA methylation status [30,31]. As the genomic information of *Triticum aestivum* is incomplete, level of cytosine methylation of wheat cannot be detected by methylome identification method, which provides a good platform for MSAP method to detect the level of methylation of *Triticum aestivum*.

Wheat is one of the most worldwide major cereal crops and its epigenetic regulation mechanism involved in DNA methylation has been proved to play crucial roles in the wheat development. As DNA methylation can repress chromosome breakages induced by the Gc factors and it can function in maintaining genome stability and regulating gene expression, we assume that they may also play a role in the Gc phenomenon. To investigate this possibility, CS–3C and CS–3C3C were used to explore the Gc chromosome molecular mechanism in wheat through identifying the DNA methylation variation of them when compared with CS. The alteration of cytosine methylation was examined by MSAP technique. Through analyzing the levels and patterns of methylation in common wheat CS, CS–3C and CS–3C3C, sequencing the differentially expressed MSAP fragments and exploring their homology, we attempt to reveal whether DNA methylation could play a role in the Gc action.

## 2. Results

### 2.1. Increased Level of Cytosine Methylation in the CS–3C Gametocidal Chromosome Addition Lines

*Eco*R I + *Hpa* II and *Ec*oR I + *Msp* I primer combinations were used to detect DNA methylation variations at the 5′-CCGG-3′ sequences in CS, CS–3C and CS–3C3C (Table 1). In CS, the overall level of methylated CCGG sites (Cs) was 41.31%, of which 32.14% were fully internal cytosine methylated sites and 9.17% were external cytosine methylated sites. The cytosine methylation levels of CS–3C and CS–3C3C were increased compared to that of CS. In CS–3C, the overall level of methylated CCGG sites (Cs) was 48.68%, and methylation of external Cs was increased to 9.81% and internal Cs was 38.87%. While in CS–3C3C, the overall level of methylated CCGG sites (Cs) (48.65%) was equal to that of CS–3C and still higher than that of CS and internal and external cytosine methylated sites were 38.19% and 10.45%, respectively (Table 2). Statistical analysis showed that there was a significant difference in methylated level between CS–3C, CS–3C3C and CS (*p* < 0.05) while there was no significant difference between CS–3C and CS–3C3C (*p* > 0.05). These results indicated that introduction of Gc chromosome(s) can lead to increased level of cytosine methylation. Interestingly, a general trend of higher levels of fully methylated and hemimethylated bands were observed in the pattern of cytosine methylation of CS–3C Gc chromosome additions compared to CS and the fully methylated loci (CG) were more than hemimethylated (CHG) loci.

### 2.2. Alteration of Locus-Specific Cytosine Methylation Patters

**Figure 1:**
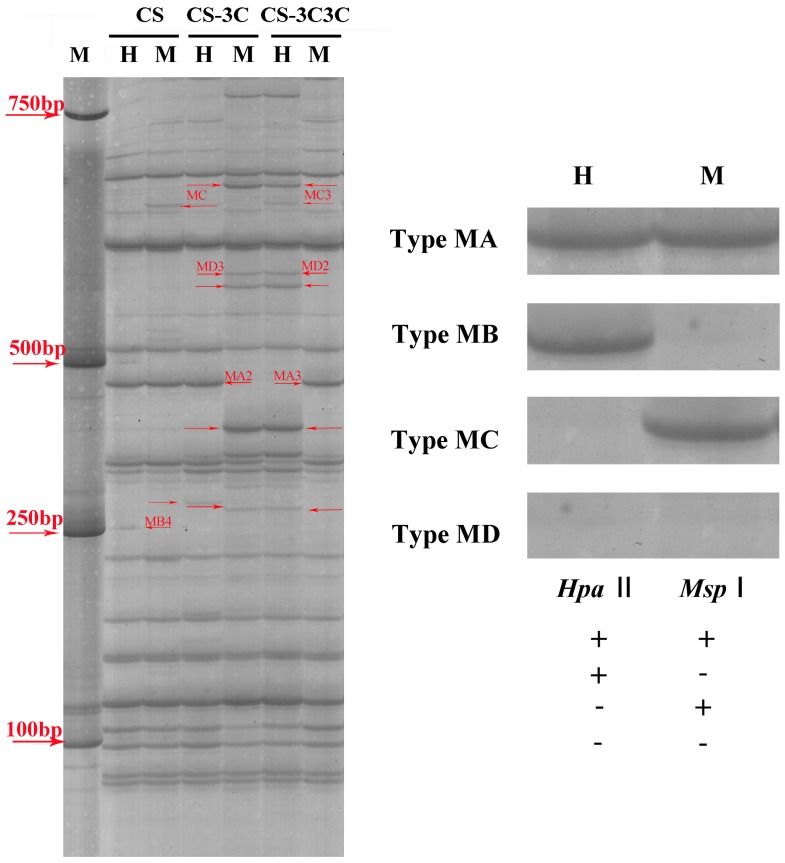
Representative variation of DNA methylation pattern. H (*Eco*R I + *Hpa* II digest) and M (*Eco*R I + *Msp* I digest) refer to digestion with *Eco*R I + *Hpa* II and *Eco*R I + *Msp* I, respectively. “→” red arrows represent parts of differential methylated bands between common wheat and wheat carrying Gc chromosome(s). M stands for Marker DL2000. MA (1, 1), presence in both H (*Eco*R I + *Hpa* II digest) and M (*Eco*R I + *Msp* I digest) lanes; MB (1, 0), presence in H and absence in M lane; MC (0, 1), absence in H but presence in M lane; MD (0, 0) absence in both H and M lanes. CS: *T. aestivum* cv. Chinese Spring. CS–3C: monosomic addition line of Chinese Spring (CS) that carries a gametocidal chromosome 3C originated from *Aegilops triuncialis*. CS–3C3C: disomic addition line of Chinese Spring (CS) that carries two gametocidal chromosome 3C originated from *Aegilops triuncialis*.

**Figure 2:**
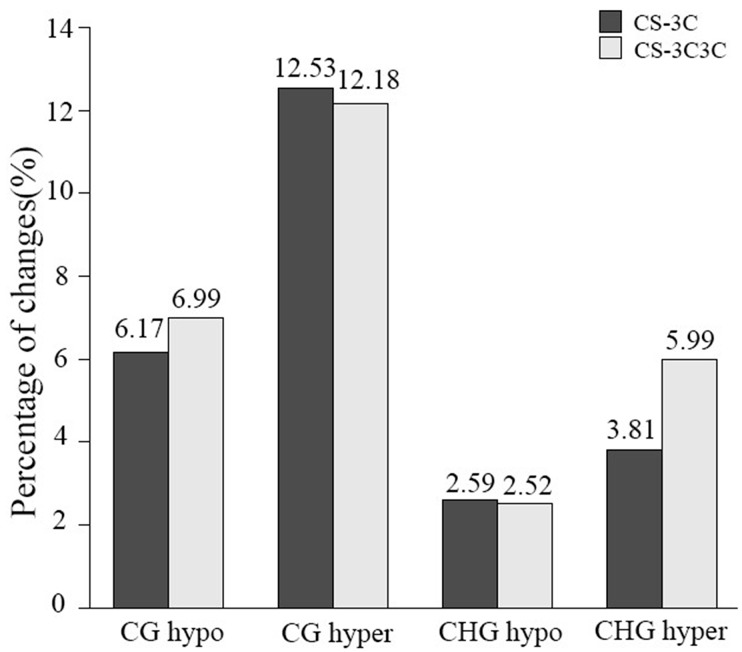
MSAP profiles showing the four patterns of cytosine methylation alterations between the CS–Gc addition lines and common wheat CS.

MSAP method can also be used to compare the modification pattern of genomes. By MSAP method, we found that various forms of changes occurred in the methylation patterns (Figure 1). Moreover, some CCGG sites of CS–3C and CS–3C3C showed the variation from full methylation to hemimethylation or in turn when compared with those of CS. The MSAP loci were divided into four major types based on presence or absence of bands due to the differential sensitivity to DNA methylation status of *Hpa* II and *Msp* I. Furthermore, the four major types could be divided into several sub-types (see Materials and Methods), according to the band types of Gc addition lines after DNA amplification. Among 16 sub-types, 73.31% and 70.76% locus including MA1-tpye, MB1-type, MC1-type and MD4-type (Table 3) showed that there existed a certain genetic similarity among CS–3C, CS–3C3C and CS during *Eco*R I/*Hpa* II and *Eco*R I/*Msp* I amplifications, respectively, and the rest displayed different genetic variation. As Figure 2 exhibited, the percentage of hypermethylated bands of CS–3C and CS–3C3C were 16.34% and 18.17%, while the percentage of hypomethylated bands of CS–3C and CS–3C3C accounted for 8.76% and 9.51%, respectively, which suggesting relatively more DNA methylation events occurred in the CS–3C Gc addition lines than in the common wheat CS. In addition, we also identified that CG hypermethylation accounted for a higher proportion than CHG hypermethylation, which suggested that cytosine methylation pattern of CG was more frequent than that of CHG, and hypermethylation of CG contributed to the increase of DNA methylation in the CS–3C Gc addition lines.

### 2.3. Sequencing Analysis of the Differentially Methylated DNA Sequences

To obtain more information of those fragments that showed differentially DNA methylated or demethylated patterns, 30 MASP bands were excised from the polyacrylamide gels and cloned into pMD^®^18-T vector, and then sequenced. Based on Blastn analysis at the NCBI website (http://blast.ncbi.nlm.nih.gov), 8 fragments showed significant homology to known sequences, of which three were homologous to MITE transposon, LTR-retrotransposon *WIS-1p* and retrotransposon *Gypsy*, respectively, and others were homologous to the *Triticum aestivum* chromosome 3B, and *Hordeum vulgare* mRNA for predicted protein (Table 4).

## 3. Discussion

DNA methylation in the form of cytosine methylation was proposed as an ancient evolutionary device [32], which has contributed to genome evolution and plays an important role in maintaining genome integrity and controlling dynamics of gene activity [12,33,34]. As reported in several plant genomes, the MSAP technique can be used as a highly efficient and reliable method for large-scale detection of cytosine methylation [35,36,37,38]. Through use of this method, our results indicated that (1) overall level of methylated CCGG sites in two Gc addition lines were significantly higher than that of the CS line; (2) various forms of alterations occurred in the methylation patterns, including hyper- and hypomethylation as well as inter-conversion of methylation types (the variation from full methylation to hemimethylation or in turn) and these alterations could occur at various genomic loci in the Gc chromosome addition lines; (3) according to the variation in the Gc chromosome addition lines, the altered patterns can be divided into several distinct groups and subgroups; and (4) based on sequence homology, the loci underwent methylation alterations in the Gc chromosome addition lines are diverse, including three transposon/retrotransposon-related sequences, MITE transposon, LTR-retrotransposon *WIS-1p* and retrotransposon *Gypsy*.

In this study, the DNA methylation levels on the ′CCGG/GGCC′ sites in CS–3C genome (48.68%) and CS–3C3C genome (48.65%) were obviously higher than that of CS genome (41.31%), and the methylation patterns on different sites exhibited rich polymorphisms, in which some sites exhibited demethylation, other sites exhibited hypermethylation. It has been suggested that the Gc chromosomes have two functions: “breaking” and “protecting” functions. Some studies have reported that treatment with the demethylation reagent 5-azacytidine can enhance chromosome aberration in common wheat carrying the Gc chromosome and it seems that DNA methylation can repress chromosome breakages induced by the Gc factors that is DNA methylation prevented the role of “breaking” in Gc mechanism [13,14]. Thus, we speculated that the sites which exhibited hypomethylation maybe participate in “breaking” function and other sites which exhibited hypermethylation maybe participate in “protecting” function. Furthermore, as the results indicated that the percentage of hypermethylated bands of CS–3C and CS–3C3C is higher than the percentage of hypomethylation bands, we speculated that DNA methylation on the ′CCGG/GGCC′ sites maybe mainly participate in “protecting” functions, especially through the CG methylation, for CG hypermethylation accounted for a higher proportion than CHG hypermethylation.

Transposable elements (TEs) are defined as discrete DNA sequences in the genome that are able to multiply and/or move within the genome [39,40]. TEs have been identified in all organisms analyzed, with similarities among kingdoms and can occupy a high proportion of a species’ genome, for example, transposable elements comprise approximately 90% of the wheat genome [41,42]. TEs as important contributors to genomic organization and major drivers of genome evolution are important for plant [39]. However, active TEs are potential harmful to the host, for example, TEs mobilization can induce illegitimate recombination, chromosome breakage and genome rearrangement [43,44]. Thus, the host and TEs have develop a series of strategies to minimize the impact of transposition both at transcription and after transcription, for example, TEs can be silenced by DNA methylation at transcription [39,43]. In our study, the sequenced results (Table 4) indicated that three polymorphism fragments were homologous to MITE transposon, LTR-retrotransposon *WIS-1p* and retrotransposon *Gypsy*, respectively. The MSAP results proved that the ‘CCGG/GGCC’ sites on MITE transposon were hypomethylation in CS–3C while the ‘CCGG/GGCC’ sites on LTR-retrotransposon *WIS-1p* and retrotransposon *Gypsy* were hypermethylation. As reported, TEs can be silenced by DNA methylation at transcription and Martínez et al. had found that hypomethylation of LINE-1 is associated with centromeric instability and maybe further induce the chromosomal instability [39,43,45]. Thus, we supposed that the Gc gene would activate some specific transposons, like MITE transposon, by reducing the level of DNA methylation and the active transposons would lead to TE mobility and they may move to other specific positions within the genome and induce chromosome breakage unless they were protected by methylation, this process might have resulted in the “breaking” function of Gc gene. However, some special transposons like LTR-retrotransposon *WIS-1p* and retrotransposon *Gypsy* may be silence as a result of being methylated and other specific positions may be also protected by being methylated, which maybe function as the “protecting” function. Based on the hypothesis above, we proposed a possible mechanism about the interaction among Gc chromosomes, DNA methylation and transposons. The Gc chromosome monosomic addition lines would produce two kinds of gametes, with or without the Gc chromosome. The active transposons like MITE transposon may move into the gametes without Gc chromosome and insert into some specific positions and finally induce chromosome breakage, however, in the gametes with the Gc chromosome some specific position could be protected by being DNA methylated and ensure the stable heredity of Gc chromosomes. In the Gc chromosome disomic addition line, all the gametes have the Gc chromosome, they could all activate these specific transposons and at the same time they could all protect specific position by being DNA methylated and these changes were balance. This can explain why there was no significant difference in the overall level of methylated CCGG sites between CS–3C and CS–3C3C. Furthermore, as the levels of cytosine methylation in both CS–3C and CS–3C3C were significant higher than that of CS, we speculated that DNA methylation on the ′CCGG/GGCC′ sites maybe mainly participate in “protecting” functions. When the Gc chromosomes were introduced into the wheat, they maybe function their “breaking” function by other epigenetic factors such as small RNA or asymmetric CHH methylation which have been reported that can function in genome stability and gene expression to induce chromosome breakage. The increased DNA methylation on the ′CCGG/GGCC′ sites maybe overshadow the “breaking” function caused by other epigenetic factors to ensure the stable heredity of Gc chromosomes. This corresponds with the previous reports that DNA methylation can repress chromosome breakages induced by the Gc factors [13,14]. The regulation mechanism can be used to explain part of the dual-function of Gc chromosomes, at least, part of the “protecting” function and why the levels of methylation of CS–3C and CS–3C3C were higher than that of CS. However, how the Gc chromosomes to achieve this particular mechanism, and whether and which other epigenetic factors can involve in the “breaking” and “protecting” functions of Gc chromosome, especially the “breaking” function and how other epigenetic factors function remains unknown and need further research.

## 4. Materials and Methods

### 4.1. Plant Materials and Growth Conditions

Three wheat genotypes cultivars were used in this investigation. One line is common wheat, *T. aestivum* cv. Chinese Spring (CS, AABBDD, 21 II, 2*n* = 42) and another two are monosomic addition line of Chinese Spring (CS) that carries a Gc chromosome 3C originated from *Aegilops triuncialis* (CS–3C, AABBDD + 3C I, 21 II + 1 I, 2*n* = 43) and disomic addition line of Chinese Spring (CS) that carries two Gc chromosome 3C originated from *Aegilops triuncialis* (CS–3C3C, AABBDD + 3C II, 22 II, 2*n* = 44). Cultivars CS seeds were obtained from the National BioResource Project. Seeds of CS–3C was obtained by hybridization from CS and CS–Gc chromosomes 3C disomic addition line (CS–3C3C), seeds of which were provided by Endo from Kyoto University in Japan. The seeds of plants were vernalized in the dark at 4 °C and then planted in pots and thereafter maintained in a greenhouse at 18–20 °C under a 16 h/8 h light/dark photoperiod. The anthers of CS, CS–3C and CS–3C3C were collected and used for subsequent DNA extraction.

### 4.2. DNA Extraction

Genomic DNA was extracted from anthers of CS, CS–3C and CS–3C3C using DNAsecure Plant Kit (TIANGEN, DP320, Beijing, China) according to the manufacturer’s instructions. The quality and concentration of DNA sample was tested by 1% agarose gel electrophoresis and absorbance ratio of OD260/OD280 using Nano-Drop^®^ND-1000 (Nanodrop Technologies, Wilmington, DE, USA). The concentration of the various DNA samples was adjusted for consistency, and then the samples were stored at −20 °C.

### 4.3. Enzyme Cleavage and Adaptor Ligation of MSAP Analysis

To analyze the variation of DNA methylation between common wheat CS, CS–3C and CS–3C3C, MSAP method was used in this research. The Genomic DNA sample was digested by *Eco*R I/*Hpa* II and *Eco*R I/*Msp* I independently and simultaneously at 37 °C for 6 h and then ligated with *Eco*R I and *Hpa* II/*Msp* I adapters (Table 1) at 8 °C for 4 h. The digestion and ligation reaction system was in a total volume of 20 μL containing 3 μL genomic DNA, 1 μL *Eco*R I (R0101L, NEB), 1 μL *Hpa* II (R0171L, NEB) or *Msp* I (R0106L, NEB), 1 μL *Eco*R I adapter, 1 μL *Hpa* II/*Msp* I adapters, 1 μL T4 DNA ligase (R0202L, NEB), 2 μL 10× T4 buffer (R0202L, NEB) and double-distilled water to 20 μL.

### 4.4. Preselective Amplification

The pre-amplification reaction consisted of: 3 μL adaptor ligation product, both 1 μL of *Eco*R I pre-selective primer and H/M pre-selective prime, 1 μL dNTP (2.5 mM each), 2 μL 10× PCR buffer with Mg^2+^ (Takara, Dalian, China), 0.15 μL Taq DNA polymerase (Takara, Dalian, China) and double-distilled water to 20 μL. PCR conditions were as follows: 94 °C for 2 min, followed by 30 cycles of denaturation for 30 s at 94 °C, annealing for 30 s at 56 °C and extension for 60 s at 72 °C and final extension for 10 min at 72 °C to complete extension. The quality and concentration of pre-amplification products was tested by 1% agarose gel electrophoresis and then the product was stored at −20 °C. Pre-amplification product was diluted 30 times (*v*/*v*) and used as DNA template for selective amplification.

### 4.5. Selective Amplification

*Eco*R I and *Hpa* II/*Msp* I primers with three additional selective nucleotides were used in this step (Table 1). The 15 μL selective amplification reaction consisted of: 1.5 μL diluted DNA pre-amplification product, 1.5 μL 10× PCR buffer with Mg^2+^ (Takara, Dalian, China), 0.6 μL dNTP (2.5 mM each) (Takara, Dalian, China), both 0.6 μL of *Eco*R I selective primer and H/M selective primer and 0.12 μL Taq DNA polymerase (Takara, Dalian, China) and double-distilled water to 15 μL. PCR conditions were as follows: 94 °C for 2 min, followed by 10 cycles of denaturation for 30 s at 94 °C, annealing for 30 s at temperature from 65 to 55 °C (each cycle reduces in 1 °C increments) and extension for 80 s at 72 °C, follow by 35 cycles of denaturation for 30 s at 94 °C, annealing for 30 s at 55 °C and extension for 80 s at 72 °C, and final extension for 10 min at 72 °C and then the product was stored at 4 °C. Selective amplified products were denatured at 95 °C for 10 min, 8 °C for 20 min and quickly chilled on ice, then mixed with 3× loading buffer. The 8 μL denatured PCR-amplified products were separated on 5% denaturing polyacrylamide gels, running in 1× TBE buffer (Tris-Borate-EDTA ) at 55 W for about 2 h, and then visualized by silver staining.

### 4.6. MSAP Band Scoring and Data Analysis

Only clear and reproducible bands were scored for polymorphisms, where the presence of a band was scored as “1” and absence of a band was scored as “0” at the same fragment size. *Hpa* II and *Msp* I are a pair of isoschizomers that show different sensitivity to methylation at the 5’CCGG sites. *Msp* I is sensitive to methylation of the external cytosine whereas *Hpa* II will be inactive if either of the cytosines is fully methylated. Therefore, MSAP method can recognize fully methylation and hemi-methylation at CCGG sites for DNA sample. In this study, we classified the amplified sites of our materials by MSAP method into four types as described before [46,47]: MA (1, 1), presence in both H and M lanes (Figure 1), represents that the site is non-methylation; MB (1, 0), presence in H and absence in M lane, indicates that the site is methylated outside the DNA single strand, also known as hemimethylation; MC (0, 1), absence in H but presence in M lane, refers to internal cytosines (CG) of CCGG sites are fully methylated; MD (0, 0) absence in both H and M lanes, means both internal and external cytosines of CCGG sites are methylated.

According to the band types of Gc addition lines after DNA amplification, we divided the four types into more sub-types as followed (Table 3 and Figure 2): 1. CG-hyper (internal cytosine methylation is higher in CS–3C than CS) include MA3 (1, 1→0, 1) and MB4 (1, 0→0, 0); 2. CHG-hyper (external cytosine methylation is higher in CS–3C than CS) contain MA2 (1, 1→1, 0) and MC4 (0, 1→0, 0); 3. Both-hyper (both internal and external cytosine methylation are higher in CS–3C than CS) refers to MA4 (1, 1→0, 0); 4. CG-hypo (internal cytosine methylation is lower in CS–3C than CS) contain MC2 (0, 1→1, 1) and MD2 (0, 0→1, 0); 5. CHG-hypo (external cytosine methylation is lower in CS–3C than CS) include MB2 (1, 0→1, 1) and MD3 (0, 0→0, 1); 6. Both-hypo (both internal and external cytosine methylation are lower in CS–3C than CS) refers to MD1 (0, 0→1, 1).

### 4.7. Cloning and Sequencing of MSAP Fragments

Bands of interest in the MSAP gel were excised and re-amplified with appropriate primers under the same conditions. Sizes of the PCR products were verified by agarose gel electrophoresis and then the PCR products were cloned into the pMD^®^18-T Vector (Takara, Dalian, China) and sequenced. Advanced Blastn programs on the NCBI website (https://blast.ncbi.nlm.nih.gov/Blast.cgi) were respectively used for mapping and homology analysis of the cloned DNA sequences that gave quality-reads.

## Figures and Tables

**Table 1:** Primers and adapters used for methylation-sensitive amplified polymorphism (MSAP).

Primer/Adapter	*Eco*R I (*E*) 5′-3′	*Hpa* II/*Msp* I (*H/M*) 5′-3′
Adapter-1	CTCGTAGACTGCGTACC	GACGATGAGTCTAGAA
Adapter-2	AATTGGTACGCAGTCTAC	CGTTCTAGACTCATC
Pre-selective primers	GACTGCGTACCAATTCA	GATGAGTCTAGAACGGT
Selective amplification primers	GACTGCGTACCAATTC AAC(Ea)	GATGAGTCTAGAACGG TAC(H/M1)
	GACTGCGTACCAATTC AAG(Eb)	GATGAGTCTAGAACGG TAG(H/M2)
	GACTGCGTACCAATTC ACA(Ec)	GATGAGTCTAGAACGG TCT(H/M3)
	GACTGCGTACCAATTC ACT(Ed)	GATGAGTCTAGAACGG TCG(H/M4)
	GACTGCGTACCAATTC ACC(Ee)	GATGAGTCTAGAACGG TTC(H/M5)
	GACTGCGTACCAATTC ACG(Ef)	GATGAGTCTAGAACGG TTG(H/M6)
	GACTGCGTACCAATTC AGC(Eg)	GATGAGTCTAGAACGG TTA(H/M7)
	GACTGCGTACCAATTC AGG(Eh)	GATGAGTCTAGAACGG TGA(H/M8)
	GACTGCGTACCAATTC AGA(Ei)	GATGAGTCTAGAACGG TGC(H/M9)
	GACTGCGTACCAATTC ATC(Ej)	GATGAGTCTAGAACGG TGT(H/M10)

**Table 2:** Patterns of genomic DNA cytosine methylation in anthers of CS, CS–3C and CS–3C3C.

Cultivars	Total Amplified Sites	Unmethylated Sites and Ratio	Methylated Sites		
			Full Methylated Sites (CG) and Ratio	Hemimethylated Sites (CHG) and Ratio	Total Methylated Sites and MSAP Ratio
CS	2956	1735 (58.69%)	950 (32.14%)	271 (9.17%)	1221 (41.31%)^b^
CS–3C	2956	1517 (51.32%)	1149 (38.87%)	290 (9.81%)	1439 (48.68%)^a^
CS–3C3C	2956	1518 (51.35%)	1129 (38.19%)	309 (10.45%)	1438 (48.65%)^a^

The total amplified sites = MA + MB + MC + MD. Full methylated ratio (%) = ((MC + MD)/(MA + MB + MC + MD)) × 100. Hemi-methylated ratio (%) = ((MB)/(MA + MB + MC + MD)) × 100. MSAP (%) = ((MB + MC + MD)/(MA + MB + MC + MD)) × 100. MA (1, 1), presence in both H (*Eco*R I + *Hpa* II digest) and M (*Eco*R I + *Msp* I digest) lanes; MB (1, 0), presence in H and absence in M lane; MC (0, 1), absence in H but presence in M lane; MD (0, 0) absence in both H and M lanes. The test used was the Duncan’s multiple range test. The different letters indicate significant difference (*p* < 0.05), while the same letters indicate no significant differences (*p* > 0.05). CS: *T. aestivum* cv. Chinese Spring. CS–3C: monosomic addition line of Chinese Spring (CS) that carries a gametocidal chromosome 3C originated from *Aegilops triuncialis*. CS–3C3C: disomic addition line of Chinese Spring (CS) that carries two gametocidal chromosome 3C originated from *Aegilops triuncialis*.

**Table 3:** Comparison of DNA methylation patterns between the CS–Gc addition lines and common wheat CS.

Patterns	Common Wheat		Gc Addition Line		Number and Frequency of Sites		Sataus
	H	M	H	M	CS–3C	CS–3C3C	
MA1	+	+	+	+	1324 (44.79%)	1304 (44.11%)	→
MA2	+	+	+	-	68 (2.30%)	86 (2.91%)	↑
MA3	+	+	-	+	306 (10.35%)	273 (9.24%)	↑
MA4	+	+	-	-	37 (1.25%)	72 (2.44%)	↑
MB1	+	-	+	-	164 (5.55%)	171 (5.78%)	→
MB2	+	-	+	+	37 (1.25%)	28 (0.95%)	↓
MB3	+	-	-	+	24 (0.81%)	21 (0.71%)	others
MB4	+	-	-	-	46 (1.56%)	51 (1.73%)	↑
MC1	-	+	-	+	652 (22.06%)	587 (19.86%)	→
MC2	-	+	+	+	139 (4.70%)	173 (5.85%)	↓
MC3	-	+	+	-	23 (0.78%)	25 (0.85%)	others
MC4	-	+	-	-	26 (0.88%)	55 (1.86%)	↑
MD1	-	-	+	+	17 (0.58%)	13 (0.44%)	↓
MD2	-	-	+	-	35 (1.18%)	27 (0.91%)	↓
MD3	-	-	-	+	31 (1.05%)	40 (1.35%)	↓
MD4	-	-	-	-	27 (0.91%)	30 (1.01%)	→

Column H: pattern after digestion with *Eco*R I and *Hpa* II; Column M: pattern after digestion with *Eco*R I and *Msp* I. “↑” increased methylation, “↓”decreased methylation, “→” no methylation changes, “others” uncertain DNA methylation.

**Table 4:** BLAST (Basic Local Alignment Search Tool) results of eight randomly selected polymorphic methylated fragments.

MSAP Fragment	Primer Combination	Length (bp)	Methylation Pattern	Accession No.	e Value	Sequence Homology
P1	Ef/HM1	240	Demethylated	HG670306.1	1×10^−74^	*Triticum aestivum* chromosome 3B, genomic scaffold, cultivar Chinese Spring
P2	Ee/HM4	247	Demethylated	AK375691.1	6×10^−32^	*Hordeum vulgare* subsp. vulgare mRNA for predicted protein, complete cds, clone: NIASHv3101H12
P5	Ee/HM4	140	Demethylated	AY534123.1	3×10^−32^	*Aegilops tauschii* transposons Stowaway MITE, transposons XJ1, Jody, Angela, and XJ and Stowaway MITE, complete sequence
P12	Ee/HM4	103	Methylated	DQ537335.1	2×10^−25^	*Triticum aestivum* clones BAC 1031P08; BAC 754K10; BAC 1344C16, complete sequence (transposon:LTR-retrotransposon *WIS-1p*)
P14	Ee/HM4	100	Methylated	HG670306.1	4×10^−25^	*Triticum aestivum* chromosome 3B, genomic scaffold, cultivar Chinese Spring
P16	Ee/HM4	240	Demethylated	HG670306.1	4×10^−78^	*Triticum aestivum* chromosome 3B, genomic scaffold, cultivar Chinese Spring
P21	Ee/HM4	137	Demethylated	HG670306.1	3×10^−23^	*Triticum aestivum* chromosome 3B, genomic scaffold, cultivar Chinese Spring
P28	Ee/HM4	143	Methylated	JF946485.1	1×10^−5^	*Triticum aestivum* retrotransposons *Gypsy* TREP 3245_Sabrina, Copia TREP 3161_WIS, complete sequence

## References

[1] Endo T. (1990). Gametocidal chromosomes and their induction of chromosome mutations in wheat. Jpn. J. Genet..

[2] Endo T.R., Tsunewaki K. (1975). Sterility of common wheat with *Aegilops triuncialis* cytoplasm. J. Hered..

[3] Tsujimoto H., Tsunewaki K. (1985). Gametocidal genes in wheat and its relatives. II. Suppressor of the chromosome 3c gametocidal gene of *Aegilops triuncialis*. Can. J. Genet. Cytol..

[4] Nasuda S., Friebe B., Gill B.S. (1998). Gametocidal genes induce chromosome breakage in the interphase prior to the first mitotic cell division of the male gametophyte in wheat. Genetics.

[5] King I.P., Laurie D.A. (1993). Chromosome damage in early embryo and endosperm development in crosses involving the preferentially transmitted 4S^l^ chromosome of *Aegilops sharonensis*. Heredity.

[6] Endo T.R., Yamamoto M., Mukai Y. (1994). Structural changes of rye chromosome 1R induced by a gametocidal chromosome. Jpn. J. Genet..

[7] Shi F., Endo T.R. (1997). Production of wheat-barley disomic addition lines possessing an *Aegilops cylindrica* gametocidal chromosome. Genes Genet. Syst..

[8] Li J., Xu X., Xu P., Guo C. (2003). Inducing chromosome translocation and deletions by Chinese Spring-Agilops 2C disomic addition x Chinese Spring-Elytriga 5E disomic addition. Yi Chuan Xue Bao.

[9] Liu W., Guo Y., Wu J., Wang X., Wang R., Yang X., Xu X., Li J., Li L. (2007). Cytological characteristics of F1 hybrids between wheat-agropyron addition lines and wheat-gametocidal chromosome addition line. J. Artic..

[10] Endo T. (2007). The gametocidal chromosome as a tool for chromosome manipulation in wheat. Chromosome Res..

[11] Tsujimoto H. (2005). Gametocidal genes in wheat as the inducer of chromosome breakage. Wheat Inf. Serv..

[12] Chan S.W., Henderson I.R., Jacobsen S.E. (2005). Gardening the genome: DNA methylation in *Arabidopsis thaliana*. Nat. Rev. Genet..

[13] De Las Heras J.I., King I.P., Parker J.S. (2001). 5-azacytidine induces chromosomal breakage in the root tips of wheat carrying the cuckoo chromosome 4S^l^ from *Aegilops sharonensis*. Heredity.

[14] Su W., Cong W., Shu Y., Wang D., Xu G., Guo C. (2013). Gametocidal chromosomes enhancing chromosome aberration in common wheat induced by 5-azacytidine. Genet. Mol. Res. GMR.

[15] Goll M.G., Bestor T.H. (2005). Eukaryotic cytosine methyltransferases. Annu. Rev. Biochem..

[16] Le T.N., Schumann U., Smith N.A., Tiwari S., Au P.C., Zhu Q.H., Taylor J.M., Kazan K., Llewellyn D.J., Zhang R. (2014). DNA demethylases target promoter transposable elements to positively regulate stress responsive genes in Arabidopsis. Genome Biol..

[17] Meyer P. (2011). DNA methylation systems and targets in plants. FEBS Lett..

[18] Henderson I.R., Jacobsen S.E. (2007). Epigenetic inheritance in plants. Nature.

[19] Law J.A., Jacobsen S.E. (2010). Establishing, maintaining and modifying DNA methylation patterns in plants and animals. Nat. Rev. Genet..

[20] Paszkowski J., Whitham S.A. (2001). Gene silencing and DNA methylation processes. Curr. Opin. Plant. Biol..

[21] Tycko B. (1997). DNA methylation in genomic imprinting. Mutat. Res..

[22] Furner I.J., Matzke M. (2011). Methylation and demethylation of the Arabidopsis genome. Curr. Opin. Plant Biol..

[23] Ruiz-García L., Cervera M.T., Martínez-Zapater J.M. (2005). DNA methylation increases throughout Arabidopsis development. Planta.

[24] Chen X., Hu J., Zhang H., Ding Y. (2014). DNA methylation changes in photoperiod-thermo-sensitive male sterile rice pa64s under two different conditions. Gene.

[25] Zhong L., Xu Y.-H., Wang J.-B. (2009). DNA-methylation changes induced by salt stress in wheat *Triticum Aestivum*. Afr. J. Biotechnol..

[26] Bednarek P.T., Orlowska R., Niedziela A. (2017). A relative quantitative methylation-sensitive amplified polymorphism (MSAP) method for the analysis of abiotic stress. BMC Plant Biol..

[27] Ci D., Song Y., Du Q., Tian M., Han S., Zhang D. (2016). Variation in genomic methylation in natural populations of populus simonii is associated with leaf shape and photosynthetic traits. J. Exp. Bot..

[28] Cao X., Fan G., Deng M., Zhao Z., Dong Y. (2014). Identification of genes related to paulownia witches’ broom by AFLP and MSAP. Int. J. Mol. Sci..

[29] Gao R., Wang H., Dong B., Yang X., Chen S., Jiang J., Zhang Z., Liu C., Zhao N., Chen F. (2016). Morphological, genome and gene expression changes in newly induced autopolyploid *Chrysanthemum lavandulifolium* (fisch. Ex trautv.) makino. Int. J. Mol. Sci..

[30] Pérez-Figueroa A. (2013). Msap: A tool for the statistical analysis of methylation-sensitive amplified polymorphism data. Mol. Ecol. Resour..

[31] McClelland M., Nelson M., Raschke E. (1994). Effect of site-specific modification on restriction endonucleases and DNA modification methyltransferases. Nucleic Acids Res..

[32] Colot V., Rossignol J.L. (1999). Eukaryotic DNA methylation as an evolutionary device. Bioessays.

[33] Rangwala S.H., Richards E.J. (2004). The value-added genome: Building and maintaining genomic cytosine methylation landscapes. Curr. Opin. Genet. Dev..

[34] Takeda S., Paszkowski J. (2006). DNA methylation and epigenetic inheritance during plant gametogenesis. Chromosoma.

[35] Portis E., Acquadro A., Comino C., Lanteri S. (2004). Analysis of DNA methylation during germination of pepper (*Capsicum annuum* L.) seeds using methylation-sensitive amplification polymorphism (MSAP). Plant Sci..

[36] Xiong L., Xu C., Maroof M.S., Zhang Q. (1999). Patterns of cytosine methylation in an elite rice hybrid and its parental lines, detected by a methylation-sensitive amplification polymorphism technique. Mol. Gen. Genet. MGG.

[37] Ashikawa I. (2001). Surveying CpG methylation at 5′-CCGG in the genomes of rice cultivars. Plant Mol. Biol..

[38] Cervera M.-T., Ruiz-Garcia L., Martinez-Zapater J. (2002). Analysis of DNA methylation in *Arabidopsis thaliana* based on methylation-sensitive AFLP markers. Mol. Genet. Genom..

[39] Cantu D., Vanzetti L.S., Sumner A., Dubcovsky M., Matvienko M., Distelfeld A., Michelmore R.W., Dubcovsky J. (2010). Small RNAs, DNA methylation and transposable elements in wheat. BMC Genom..

[40] Sabot F., Simon D., Bernard M. (2004). Plant transposable elements, with an emphasis on grass species. Euphytica.

[41] Flavell R. (1986). Repetitive DNA and chromosome evolution in plants. Philos. Trans. R. Soc. Lond. B Biol. Sci..

[42] Feschotte C., Jiang N., Wessler S.R. (2002). Plant transposable elements: Where genetics meets genomics. Nat. Rev. Genet..

[43] Muñoz-López M., García-Pérez J.L. (2010). DNA transposons: Nature and applications in genomics. Curr. Genom..

[44] Slotkin R.K., Martienssen R. (2007). Transposable elements and the epigenetic regulation of the genome. Nat. Rev. Genet..

[45] Martínez J.G., Pérez-Escuredo J., Castro-Santos P., Marcos C.Á., Pendás J.L.L., Fraga M.F., Hermsen M.A. (2012). Hypomethylation of LINE-1, and not centromeric SAT-α, is associated with centromeric instability in head and neck squamous cell carcinoma. Cell. Oncol..

[46] Marconi G., Pace R., Traini A., Raggi L., Lutts S., Chiusano M., Guiducci M., Falcinelli M., Benincasa P., Albertini E. (2013). Use of MSAP markers to analyse the effects of salt stress on DNA methylation in rapeseed (*Brassica napus* var. *Oleifera*). PLoS ONE.

[47] Bocchini M., Bartucca M.L., Ciancaleoni S., Mimmo T., Cesco S., Pii Y., Albertini E., del Buono D. (2015). Iron deficiency in barley plants: Phytosiderophore release, iron translocation, and DNA methylation. Front. Plant Sci..

